# Treatment of Patients With Femoroacetabular Impingement Syndrome Using a Pelvic Tilt–Focused Exercise Program: A Prospective Cohort

**DOI:** 10.1177/03635465261454648

**Published:** 2026-06-17

**Authors:** Graeme Hoit, Daniel B. Whelan, Tim Dwyer, Sheilah Hogg-Johnson, Jaskarndip Chahal, Aileen M. Davis

**Affiliations:** † Department of Surgery, Division of Orthopaedic Surgery, University of Toronto, Toronto, Ontario, Canada; ‡ Institute of Health Policy, Management and Evaluation, Dalla Lana School of Public Health, University of Toronto, Toronto, Ontario, Canada; § Division of Orthopaedic Surgery, Women’s College Hospital, Toronto, Ontario, Canada; ‖ University of Toronto Orthopaedic Sports Medicine, University of Toronto, Toronto, Ontario, Canada; ¶ Research and Innovation, Canadian Memorial Chiropractic College, Toronto, Ontario, Canada; # Department of Physical Therapy, University of Toronto, Toronto, Ontario, Canada; Investigation performed at St. Michael's Hospital and Women's College Hospital at the University of Toronto, Toronto, Ontario, Canada

**Keywords:** femoroacetabular impingement syndrome, nonoperative management, exercise therapy, clinical outcomes

## Abstract

**Background:**

The rates of hip arthroscopy are increasing worldwide for femoroacetabular impingement syndrome (FAIS). There is a lack of literature examining outcomes of modern nonoperative treatment of patients with FAIS.

**Purpose:**

To develop and assess the effectiveness of a pelvic tilt–focused exercise program for nonoperative management for patients with FAIS.

**Study Design:**

Cohort study; Level of evidence, 2.

**Methods:**

Eligible patients included those aged 16 to 55 years who met radiographic and clinical criteria for FAIS. Enrolled patients participated in a 10-minute standardized pelvic tilt–focused home exercise program (HipFit10) at least 4 times per week over 6 months. Self-reported adherence was tracked weekly. Patient-reported outcomes were collected at baseline and 3 and 6 months. The International Hip Outcome Tool–33 (iHOT-33) score was measured at baseline and 3 and 6 months, with the primary outcome being the iHOT-33 change score between baseline and 6 months. Secondary outcomes included the Hip Outcome Score–Activities of Daily Living (HOS-ADL), pain visual analog scale (pain VAS) score, crossover to surgical management, and complications.

**Results:**

A total of 214 patients were enrolled between August 2020 and October 2021. The mean age was 34.2 ± 10.2 years, and 121 (57%) patients were female. Within the study period, 19 of 214 (9%) patients dropped out or were lost to follow-up. By 6 months, 153 of 195 (78%) patients completed the intervention and elected to avoid surgery, and 42 of 195 (22%) patients signed up for or underwent surgery. The final iHOT-33 score was completed by 192 of 195 (98%) participants with mean improvement from baseline to 6 months of 11.7 (95% CI, 9.2 to 14.1; *P* < .001). By 6 months, 103 of 192 (54%) participants achieved the iHOT-33 minimal clinically important difference of 6.8. Significant improvements also were seen in the iHOT-33 score from baseline to 3 months (mean difference [MD], 7.6; 95% CI, 5.7 to 9.6) and from 3 months to 6 months (MD, 3.7; 95% CI, 1.8 to 5.6), as well as in the HOS-ADL (0-3 months: MD, 2.65; 95% CI, 0.8 to 4.4) and pain VAS score (0-3 months: MD, −7.7 [95% CI, −4.8 to −10.5]; 3-6 months: MD, −3.5 [95% CI, −0.6 to −6.3]). No adverse events were reported.

**Conclusion:**

In this study of patients with FAIS, pelvic tilt–focused physical therapy was utilized to improve patient function with 78% avoiding surgery. These results suggest the HipFit10 program is a safe and effective nonoperative option for patients with FAIS.

Femoroacetabular impingement syndrome (FAIS) is a painful hip condition caused by abnormal bone structure of the hip joint and impaired clearance between the femoral head-neck junction and the acetabulum.^14,16^ The overall prevalence of FAIS is estimated to be 10% to 15% of the population, most of whom are young active people, aged 20 to 45 years.^48^

Current treatment for FAIS includes both operative and nonoperative interventions. While previous clinical trials have demonstrated the effectiveness of hip arthroscopy as a treatment option for patients with FAIS,^17,27,35^ surgery does present potential risks such as nerve injury, microinstability, and deep vein thrombosis.^24^ A trial of nonoperative treatment, typically in the form of physical therapy exercise, may allow patients to significantly improve their symptoms while avoiding exposure to the risks and onerous recovery associated with surgery. Pooled results from nonoperative arms of previous trials demonstrate clinically significant improvement in patients treated with an exercise-based nonoperative regimen.^17^ With rates of hip arthroscopy increasing worldwide,^7,8,10,25,26^ effective nonoperative intervention has the ability to curb some of the increasing demand, personal health and system costs, and potentially morbidity associated with surgical care in this young active population.

The evidence for the best nonoperative treatment for FAIS continues to evolve. Some have questioned whether physical therapy protocols utilized in the nonoperative arms of the aforementioned trials were in line with current best evidence for nonoperative treatment.^2,17,20,22,23,28,29,35^ Recent sentinel papers have demonstrated improved outcomes with physical therapy exercise programs focusing on core strengthening and strength of the muscles that control pelvic tilt, rather than previous emphasis on hip abductors, adductors, and external rotators.^3,19,38^ These conclusions are strengthened by the increasing body of evidence that pelvic tilt and range of motion, and the muscles involved in these actions, are implicated in symptomatic FAIS.^38^ Moreover, each trial utilized multicomponent and individualized treatment protocols, making it difficult to discern which elements of the regimen were utilized and which were effective. There is a need for further research into the effectiveness of standardized pelvic tilt exercise–based treatment protocols for patients with FAIS.^11^

The objective of this study was to develop and assess the clinical outcomes of a pelvic tilt–focused exercise program in the nonoperative management of adult patients with FAIS.

## Methods

### Setting and Study Design

The study was a prospective study of patients with FAIS at 2 Toronto academic hospital centers (Women’s College Hospital [WCH] and St. Michael’s Hospital–Unity Health [SMH]). All patients were recruited from the outpatient practices of 3 academic orthopaedic surgeons (D.B.W., T.D., J.C.) with fellowship training in sports medicine and expertise in nonarthritic hip disorders and hip arthroscopy. Those enrolled participated in a standardized physical therapy exercise program delivered by 1 of 2 advanced practice physical therapists at SMH. The study received ethical approval from both institutions’ review panels (WCH: 2018-0154-E; SMH: 20-013) and the University of Toronto (40512). This study followed the STROBE (Strengthening the Reporting of Observational Studies in Epidemiology) reporting guidelines.^45^

### Patients and Eligibility Criteria

Patients referred for FAIS were screened for eligibility by trained research associates and by the primary author (G.H.) based on established inclusion and exclusion criteria. Patients were included if aged 16 to 55 years with a clinical history in keeping with FAIS; radiographic signs of cam, pincer or mixed-type pathology; and magnetic resonance imaging (MRI) evidence of a labral tear. Radiographic parameters for inclusion included any of the following: pistol grip deformity (anteroposterior [AP] radiograph), alpha angle >55° (45° Dunn or frog-leg lateral views), lateral center-edge angle (LCEA) >40° (AP radiograph), crossover sign (AP radiograph), ischial spine sign (AP radiograph), or Tönnis angle <0° (AP radiograph).^49^

Exclusion criteria were established based on factors that are known or widely thought to affect the patients’ ability to perform exercises or influence their performance on patient-reported outcome measures (PROMs) based on the orthopaedic literature. Those with hip dysplasia, Legg-Calve-Perthes disease, slipped capital femoral epiphysis, arthritis Tönnis angle >1°, inflammatory arthritis, Ehlers-Danlos syndrome, complex regional pain syndrome, fibromyalgia, chronic opioid use, and other pain syndromes were excluded.^34,40,48^ Additionally, patients with a workplace injury claim or a medicolegal claim for a motor vehicle collision related to their hip pain were excluded.^32,34,36^

Eligible patients agreeing to participate in the study provided written consent.

### Study Procedures

After completion of baseline data collection, all participants had a visit with 1 of 2 advanced practice physical therapists, where the HipFit10 exercises (described below) were demonstrated and any required modifications were made. Patients received a telephone call by a member of the orthopaedic care team at 2 weeks to check the initiation of the program and to answer any clinical questions the patients had. A second visit with the same physical therapist was scheduled for each patient 4 weeks after their initial visit, whereby the exercises were reviewed and observed by the treating therapist. Corrections to exercise form and modifications to exercises were made as necessary.

Patients were seen in clinical follow-up by their treating surgeon at 3 months and 6 months, as per standard of care, for repeat evaluation and measurement of their PROMs as well as the collection of clinical and radiographic data. At the 3-month visit, patients were encouraged to continue with the HipFit10 program for the full 6-month duration. Those who thought they had not improved were permitted to cross over at 3 months to operative management in the form of hip arthroscopy, osteochondroplasty, and labral repair. Similarly, at the 6-month visit, patients had the option of continuing with nonoperative care or pursuing operative management.

### Intervention and Design of the HipFit10 Program

**Figure 1.**
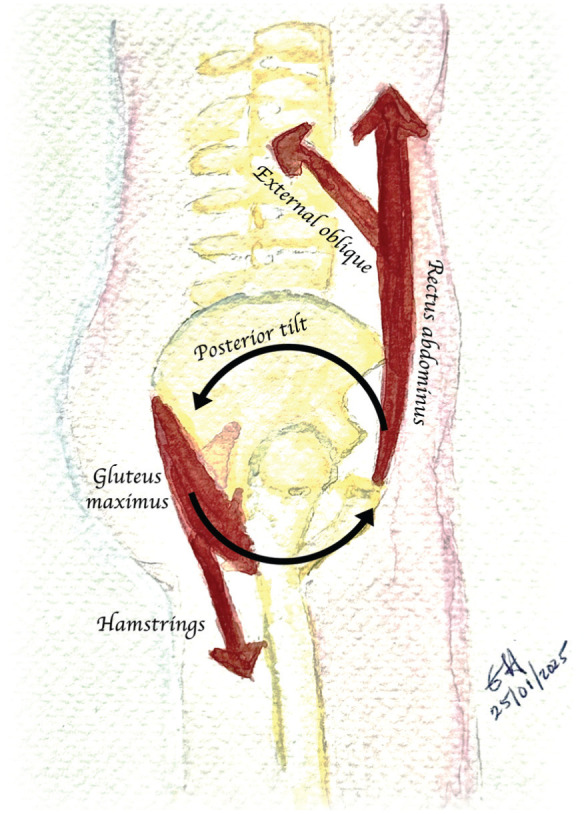
Visualization of muscles affecting pelvic tilt.

Despite consensus that exercise-based treatment for FAIS ought to be first-line for all patients, very little evidence exists guiding the creation of an exercise-based intervention.^16,22^ We conducted a systematic review and meta-analysis of all randomized trials comparing nonoperative exercise-based treatment protocols as the foundation for the creation of our intervention program.^19^ When meta-analyzed, we found that protocols focused on active strengthening led to more improvement compared with nonstrengthening modalities (Transcutaneous Electrical Nerve Stimulation [TENS], ultrasound, massage, stretching, dry needling, acupuncture, heat and cold therapy, etc), that core-based exercise demonstrated greater improvement than hip-focused therapy, and that supervised physical therapy led to more improvement than self-guided exercise.^19^ These principles were used to guide the creation of the HipFit10 program. We also consulted the guidance provided by the American College of Sports Medicine for the creation of exercise therapy programs.^2^ Based on iterative feedback from patients, we aimed to create a program that patients thought was feasible, which included being time-efficient and not requiring specialized equipment or significant travel time. This led to the selection of 10 strengthening exercises that were previously utilized in FAIS therapy protocols,^3,46^ which were low impact, extension based to avoid impingement moments, and focused on abdominal, gluteal, and hamstring muscles (see Appendix 1, available in the online version of this article). These target muscle groups were selected as they are the muscles responsible for rotating the pelvis into a posteriorly tilted position (Figure 1), avoiding anterior impingement.^38^

The patients were provided a printed handout of the exercises as well as a web-based platform (www.hipfit10.com) further detailing the exercises and answering frequently asked questions for patients viewing at home. Patients were instructed to complete the exercises 4 times weekly depending on tolerability, with off-days incorporating a stretching routine as tolerated.^5,37^ The duration of the program was described as a minimum of 3 months, with encouragement for patients to continue to 6 months.

In addition to the exercises, patients were permitted to engage in activity modification and take over-the-counter medication, including acetaminophen and nonsteroidal anti-inflammatory drugs, as needed. Patients were asked to avoid weighted strengthening exercises that focused on the antagonistic muscle pairs of the targeted groups of the HipFit10 program (namely avoiding low back, quads, and hip flexor strengthening). Beyond this, patients were not formally restricted from any fitness activities during their treatment, although they were advised to avoid high-impact activities that aggravated their hip pain for the first 3 months.

### Outcomes

The primary outcome was the change in International Hip Outcome Tool–33 (iHOT-33) score from baseline to 6 months. The proportion of patients who achieved at least the minimal clinically important difference (MCID), 6.8 points, at 3 and 6 months compared with their baseline score were also quantified.^31,42^ The iHOT-33 score is a reliable PROM of hip-related disability and quality of life validated for use in patients with FAIS,^18,31^ including those treated nonoperatively,^42^ and it has performed well in psychometric evaluations in similar patient groups.^21,44^ It consists of 33 questions, each scored on a scale of 0 to 100, where a higher score represents better quality of life. The final score is the mean of all answered questions. Secondary outcome measures included the Hip Outcome Score–Activities of Daily Living (HOS-ADL),^21^ a 17-item questionnaire scored from 0 to 100, with higher scores indicating better daily function; the pain visual analog scale (pain VAS) score, a single VAS question on a scale from 0 to 100, with higher scores indicating more pain; and the proportion of patients who avoided surgery at 3 and 6 months. Patients were asked about any complications at each follow-up interval during the treatment program, and any reported complications were recorded.

### Baseline Data Collection

Relevant descriptive, clinical, and radiographic data were collected at the initial consultation and entered into a secure REDCap database. Patient data included age, sex, and body mass index (BMI). For clinical data, patients were asked the duration of their pain and its primary location (groin pain, pain of the lateral hip overlying the greater trochanter, posterior gluteal pain, low back pain, or anterior thigh pain were options). The quality of the pain (achy, dull, sharp, burning, or shooting) and mechanical symptoms (clicking, catching, popping, or locking) was captured. Previous treatments were recorded, including joint injections and physical therapy. Physical therapy was subsequently subclassified into the type of therapy (nonstrengthening therapy such as TENS, ultrasound, massage, stretching, etc, vs strengthening therapy including peri-hip muscle strengthening [abductors, hip flexors, rotators, and adductors], gait retraining, and core muscle strengthening [abdominal muscles and gluteals]). Baseline patient activity level was captured using the modified Tegner activity score, which ranges from 0 (completely disabled) to 10 (Olympic or professional athlete).^43^

All patients enrolled in the study underwent radiographic imaging and MRI, as per standard of care.^16^ Radiograph views included AP pelvis, false-profile, and 45° Dunn lateral views of both hips. The MRI study was of the affected hip and could include 1.5-T or 3-T MRI or magnetic resonance arthrography. Imaging was evaluated at the time of initial consultation by the treating surgeon for diagnosis and patient eligibility and underwent a repeat evaluation by the primary investigator (G.H.) for study data collection. Radiograph data were measured in accordance with the same imaging protocol described by Zhou et al^49^ in their assessment of prevalence of structural deformity in patients with FAIS.

Patients were asked to complete baseline PROMs including iHOT-33, HOS-ADL, and pain VAS scores via survey links through REDCap (Table 1).

**Table 1:** Participant Timeline^*a*^

Activity/Assessment	Consultation	Initial PT Visit	2-Wk Telephone Call	4-Wk PT Visit	3-Mo FU	6-Mo FU
Study enrollment	X					
Baseline CRF and physical examination	X					
Radiographs and MRI assessment	X					
Demonstration of exercises		X		X		
Core strength assessment	X				X	X
Modified Tegner activity score	X					
iHOT-33 score	X				X	X
HOS-ADL	X				X	X
Pain VAS score	X				X	X
Opportunity to cross over to surgery					X	X

a CRF, case report form; FU, follow-up; HOS-ADL, Hip Outcome Score–Activities of Daily Living; iHOT-33, International Hip Outcome Tool–33; MRI, magnetic resonance imaging; PT, physical therapy; VAS, visual analog scale.

### Adherence Collection

Self-reported program adherence was collected on a weekly basis throughout the duration of the program. Each participant was sent a weekly REDCap survey question asking, “How many days in the past 7 days did you complete your HipFit10 exercises?” with a range of 0 to 7 available.

### Statistical Analysis and Sample Size Calculation

Baseline descriptive statistics were calculated and presented as mean with standard deviation or median with interquartile range for continuous variables, where appropriate, and relative frequency with percentage for categorical variables.

Changes in patient-reported outcomes (including the primary outcome of iHOT-33 score) across different time points were assessed using paired *t* tests. The significance threshold was set at a *P* value <.05. Missing data were excluded from outcome analysis.

The number of patients who achieved improvement as large as or larger than the MCID of the measured PROMs, as well as the number of patients who avoided surgery, was calculated and presented as count and proportion.

Primary analyses were performed on an intention-to-treat basis, regardless of patient self-reported adherence. An exploratory analysis was conducted to assess the effect of program adherence comparing patients who adhered to the program versus those who did not, with the adherence threshold being completion of exercises at least 3 times per week in at least 75% of the first 12 weeks. A post hoc subgroup analysis comparing outcomes across different impingement types (cam, pincer, and mixed) was conducted, with continuous outcomes compared using analysis of variance tests and categorical outcomes compared using chi-square tests.

The analysis for this study was generated using SAS software (Version 9.4; SAS).

A sample size calculation was conducted utilizing PROC POWER to determine the number of participants required to find significance in our primary analysis with a mean difference of 10 points^21^ between baseline and 6-month iHOT-33 scores, an alpha set to .05, and a standard deviation of 20.1^21^ with 90% power, which demonstrated that 172 patients would be required. Assuming a 20% attrition rate, a final sample of 214 patients was established as the study target.

## Results

### Participant Characteristics

**Figure 2.**
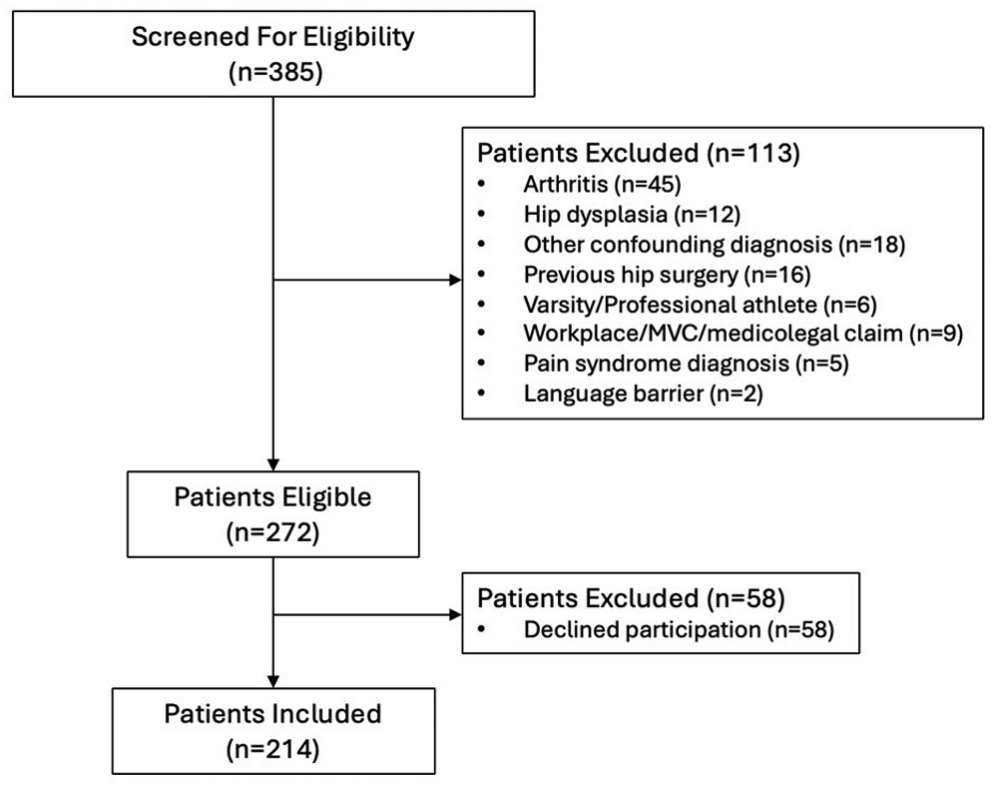
Participant diagram. MVC, motor vehicle collision.

A total of 214 patients were enrolled in the study, with 121 (56.5%) females and 93 (43.5%) males (Figure 2). The mean age of participants was 34.2 ± 10.2 years (range, 16-55 years). Participants covered a wide range of activity levels (mean Tegner score, 5.3 ± 1.8; range, 2-8) and BMI (mean, 25.4 ± 4.9 kg/m^2^; range, 16.8-43.6 kg/m^2^) (Table 2). The mean duration of symptoms was 43.5 ± 44.8 months (range, 5-240 months). A total of 81 (38%) patients reported bilateral symptoms at evaluation. The majority of participants endorsed groin pain as their primary pain location (n = 198; 93%). Most participants had engaged in physical therapy for their FAIS before enrolling in the study (n = 154; 72%); however, only 29 (14%) had participated in physical therapy that focused on core muscle strengthening. In terms of radiographic characteristics, 84 (39%) patients had cam morphology, 58 (27%) patients had pincer morphology, and 72 (34%) patients had mixed morphology, with both cam and pincer features (Table 3).

**Table 2:** Patient Characteristics^*a*^

	All Patients (N = 214)	Included Sample (n = 192)	Excluded Sample (n = 22)	Missingness
Demographics				
Age, y	34.2 ± 10.2	34.8 ± 10.1	29.0 ± 9.4	0
Female sex	121 (57)	108 (56)	13 (59)	0
BMI, kg/m^2^	25.4 ± 4.9	25.4 ± 4.7	26.3 ± 6.1	2 (1) from included sample
<18.5	4 (2)	2 (1)	2 (9)	
18.5-24.9	114 (53)	104 (54)	10 (45)	
25-29.9	58 (27)	55 (29)	3 (14)	
30-34.9	27 (13)	22 (11)	5 (23)	
35-39.9	7 (3)	5 (3)	2 (9)	
≥40	2 (1)	2 (1)	00 (0)	
Current smoking	9 (4)	8 (4)	1 (5)	0
Tegner activity score	5.3 ± 1.8	5.3 ± 1.8	5.5 ± 2.0	0
Symptoms				
Duration of symptoms, mo, median (range)	24 (5-240)	24 (5-240)	21 (5-180)	1 (0.5) from included sample
Bilateral symptoms	81 (38)	75 (39)	6 (27)	3 (1) from included sample
Groin pain	198 (93)	177 (92)	21 (95)	0
Lateral pain	62 (29)	59 (31)	3 (14)	0
Posterior pain	77 (36)	70 (36)	7 (32)	0
Thigh pain	26 (12)	22 (11)	4 (18)	0
Low back pain	21 (10)	18 (9)	3 (14)	0
Mechanical symptoms	103 (48)	89 (46)	14 (64)	
Prior treatments				
Physical therapy	154 (72)	139 (72)	15 (68)	0
Core-based PT	29 (14)	27 (14)	2 (9)	0
Nonstrengthening PT modalities	73 (34)	63 (33)	10 (45)	0
Injections	41 (19)	38 (20)	3 (14)	0
Physical examination				
FADIR	170 (79)	151 (79)	19 (86)	0
FABER	42 (20)	35 (18)	7 (32)	0
Diagnosis				
Cam	84 (39)	77 (40)	7 (32)	0
Pincer	58 (27)	49 (26)	9 (41)	0
Mixed	72 (34)	66 (34)	6 (27)	0
Patient-reported outcomes				
iHOT-33 score	42.9 ± 16.7	42.3 ± 16.2	48.4 ± 20.2	0
HOS-ADL	72.9 ± 16	72.5 ± 15.9	76.7 ± 16.6	0
Pain VAS score	53.7 ± 23.1	54.1 ± 22.7	50.3 ± 26.5	0

a Data are presented as mean ± SD or n (%) unless otherwise indicated. BMI, body mass index; FABER, flexion abduction external rotation test; FADIR, flexion adduction internal rotation test; HOS-ADL, Hip Outcome Score–Activities of Daily Living; iHOT-33, International Hip Outcome Tool–33; PT, physical therapy; VAS, visual analog scale.

**Table 3:** Radiographic Characteristics^*a*^

	All Patients	Included Sample	Excluded Sample	Missingness
All patients	N = 214 (100)	n = 192 (90)	n = 22 (10)	
Alpha angle, deg	61.5 ± 12.4	61.8 ± 12.5	58.4 ± 10.8	0
>55°	150 (70)	138 (72)	12 (55)	
>65°	94 (44)	88 (46)	6 (27)	
Pistol grip	61 (29)	55 (29)	6 (27)	0
LCEA, deg	35.4 ± 7.1	35.5 ± 7.3	35.4 ± 5.4	0
<20°	00 (0)	00 (0)	00 (0)	
>40°	60 (28)	55 (29)	5 (23)	
>50°	6 (3)	6 (3)	00 (0)	
Tönnis angle <0°	50 (23)	41 (21)	9 (41)	0
Acetabular retroversion	126 (59)	112 (58)	14 (64)	0
Spine sign	80 (37)	67 (35)	13 (59)	0
Crossover sign	101 (47)	89 (46)	12 (55)	0
Impingement cyst	77 (36)	74 (39)	3 (14)	0
Chondrosis grade 1	75 (35)	68 (36)	7 (32)	2 from included sample
Chondrosis grade 2	14 (7)	14 (7)	00 (0)	
Arthritis grade 1	64 (30)	59 (31)	5 (23)	3
Cam patients	n = 84 (39)	n = 77 (92)	n = 7 (8)	
Alpha angle, deg,	67.5 ± 10.4	67.8 ± 10.3	65.4 ± 12.1	0
>50°	77 (92)	72 (94)	5 (71)	
>65°	56 (67)	53 (69)	3 (43)	
Pistol grip	38 (45)	35 (45)	3 (43)	0
LCEA, deg	32.0 ± 5.3	32.0 ± 5.3	31.1 ± 4.7	0
Impingement cyst	41 (49)	39 (51)	2 (29)	0
Chondrosis grade 1	29 (35)	28 (37)	1 (14)	1 from included sample
Chondrosis grade 2	8 (10)	8 (11)	00 (0)	
Arthritis grade 1	29 (35)	29 (38)	00 (0)	0
Pincer patients	n = 58 (27)	n = 49 (84)	n = 9 (16)	
Alpha angle, deg	47.4 ± 6.2	47.1 ± 6.3	49.3 ± 5.7	0
LCEA, deg	38.7 ± 7.5	39.0 ± 7.9	37.2 ± 5.4	0
>40°	27 (47)	24 (49)	3 (33)	0
>50°	5 (9)	5 (10)	00 (0)	0
Tönnis angle <0°	22 (38)	17 (35)	5 (56)	0
Acetabular retroversion	43 (74)	35 (71)	8 (89)	0
Spine sign	30 (52)	23 (47)	7 (78)	0
Crossover sign	41 (71)	33 (67)	8 (89)	0
Impingement cyst	8 (14)	8 (16)	00 (0)	0
Chondrosis grade 1	19 (33)	16 (33)	3 (33)	1 from included sample
Chondrosis grade 2	1 (2)	1 (2)	00 (0)	
Arthritis grade 1	15 (26)	13 (27)	2 (22)	1
Mixed patients	n = 72 (34)	n = 66 (92)	n = 6 (8)	
Alpha angle, deg	65.7 ± 9.0	65.8 ± 9.3	64.8 ± 4.1	0
>50°	67 (93)	61 (92)	6 (100)	
>65°	38 (53)	35 (53)	3 (50)	
Pistol grip	22 (31)	20 (30)	2 (33)	0
LCEA, deg	36.9 ± 6.9	36.8 ± 7.1	37.5 ± 3.7	0
>40°	29 (40)	27 (41)	2 (33)	
>50°	1 (1)	1 (2)	00 (0)	
Tönnis angle <0°	19 (26)	16 (24)	3 (50)	0
Acetabular retroversion	60 (83)	55 (83)	5 (83)	0
Spine sign	36 (50)	31 (47)	5 (83)	0
Crossover sign	49 (68)	45 (68)	4 (67)	0
Impingement cyst	28 (39)	27 (41)	1 (17)	0
Chondrosis grade 1	27 (38)	24 (36)	3 (50)	0
Chondrosis grade 2	5 (7)	5 (8)	00 (0)	
Arthritis grade 1	20 (29)	17 (27)	3 (50)	2 from included sample

a Data are presented as mean ± SD or n (%). LCEA, lateral center-edge angle.

### Clinical Outcomes

**Figure 3.**
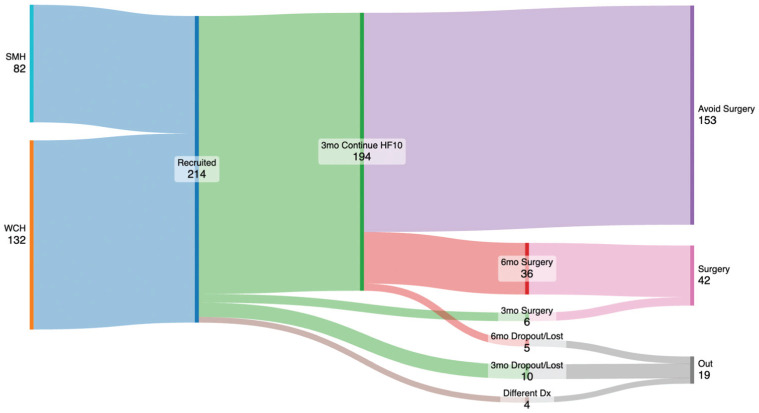
Sankey Diagram of participant tracking and outcomes. Dx, diagnosis; HF10, HipFit10 program; SMH, St. Michael’s Hospital–Unity Health; WCH, Women’s College Hospital.

In total, 19 (9%) patients from the originally enrolled 214 either dropped out (n = 9; 4%) were lost to follow-up (n = 6; 3%) or were assigned a different diagnosis at the 3-month visit (n = 4; 2%) (Figure 3). Of the remaining 195 patients, 42 (22%) elected to undergo hip arthroscopy within the 6-month study period, while 153 (78%) patients were able to avoid surgery and continue to nonoperative management after 6 months.

Of the 195 patients who remained in the study to 6 months, 192 (98%) completed their 6-month outcome questionnaires and were included in the final sample (Table 2) for outcome analysis. Their iHOT-33 scores (primary outcome) improved from baseline to 6 months (mean difference [MD], 11.7; 95% CI, 9.2 to 14.1; *P* < .001) (Table 4). Significant improvements were seen in the iHOT-33 score from baseline to 3 months (MD, 7.6; 95% CI, 5.7 to 9.6; *P* < .001), and the scores continued to improve from 3 months to 6 months (MD, 3.7; 95% CI, 1.8 to 5.6; *P* < .001). By 6 months, 103 of 192 (54%) participants achieved the iHOT-33 MCID of 6.8, including 34 (18%) participants who had not yet met the MCID by 3 months (Table 5). The HOS-ADL also improved from baseline to 3 months and 6 months, but there was no statistically significant difference between 3- and 6-month scores (0-3 months: MD, 2.7 [95% CI, 0.8 to 4.4; *P* = .005]; 0-6 months: MD, 4.2 [95% CI, 2.1 to 6.3; *P* < .001]; 3-6 months: MD, 1.5 [95% CI, −0.3 to 3.3; *P* = .11]). The pain VAS score improved from baseline to 3 months and 6 months with statistically significant improvements between 3- and 6-month scores as well (0-3 months: MD, −7.7 [95% CI, −4.8 to −10.5; *P* < .001]; 0-6 months: MD, −11.7 [95% CI, −8.4 to −15.0; *P* < .001]; 3-6 months: MD, −3.5 [95% CI, −0.6 to −6.3; *P* = .019]).

**Table 4:** Patient-Reported Outcomes^*a*^

PROM	Baseline (N = 214)^*b*^	3 Mo (n = 195)^*b*^	6 Mo (n = 192)^*b*^	Baseline to 3 Mo (n = 195)			Baseline to 6 Mo (n = 192)			3 to 6 Mo (n = 184)		
				MD	95% CI	*P* Value	MD	95% CI	*P* Value	MD	95% CI	*P* Value
iHOT-33 score	42.9 ± 16.7	50.2 ± 20.5	54.2 ± 22.0	7.6	5.7 to 9.6	**<.001**	11.7	9.2 to 14.1	**<.001**	3.7	1.8 to 5.6	**<.001**
HOS-ADL	72.9 ± 16.0	75.2 ± 16.6	77.1 ± 17.1	2.7	0.8 to 4.4	.**005**	4.2	2.1 to 6.3	**<.001**	1.5	−0.3 to 3.3	.11
Pain VAS score^*c*^	53.7 ± 23.1	46.4 ± 24.8	41.9 ± 25.4	−7.7	−4.8 to −10.5	**<.001**	−11.7	−8.4 to −15.0	**<.001**	−3.5	−0.6 to −6.3	.**019**

a Boldface type indicates statistical significance. HOS-ADL, Hip Outcome Score–Activities of Daily Living; iHOT-33, International Hip Outcome Tool–33; MD, mean difference; VAS, visual analog scale; PROM, patient-reported outcome measure.

b Data are presented as mean ± SD.

c Because of direction of scaling, negative MD represents improvement.

**Table 5:** Achievement of iHOT-33 MCID by 3 and 6 Months^*a*^

6 Mo (n = 192)	3 Mo (n = 195)			
iHOT-33	MCID−	MCID+	Missing 3 Mo	Total
MCID−	71 (39)	13 (7)	5	89/192 (46)
MCID+	34 (18)	66 (36)	3	103/192 (54)
Missing 6 mo	10	1		
Total	115/195 (59)	80/195 (41)		

a iHOT-33, International Hip Outcome Tool–33; MCID, minimal clinically important difference.

Of the patients in our adherence-based sensitivity analysis, 125 of 192 (65%) patients met the criteria for exercise adherence. As shown in Table 6, there was a trend toward a greater improvement in PROMs among those who adhered compared with those who did not, although the difference was not statistically significant (0- to 3-month iHOT-33 score: MD, 3.6; 95% CI, −0.4 to 7.5; *P* = .07).

**Table 6:** Exploratory Analysis of Outcomes by Program Adherence^*a*^

PROM	Adhered (n = 125)^*b*^	Nonadhered (n = 67)^*b*^	MD (95% CI)	*P* Value
Baseline to 3 mo				
Change in iHOT-33 score	9.0 ± 14.3	5.4 ± 12.2	3.6 (−0.4 to 7.5)	.07
Change in HOS-ADL	3.3 ± 12.0	1.6 ± 4.0	1.7 (−2.0 to 5.5)	.36
Change in pain VAS score	−8.9 ± 21.4	−5.8 ± 17.7	−3.1 (−9.0 to 2.8)	.29
Baseline to 6 mo				
Change in iHOT-33 score	12.9 ± 18.1	9.5 ± 15.0	3.4 (−1.8 to 8.5)	.18
Change in HOS-ADL	4.3 ± 13.8	4.0 ± 14.8	0.3 (−4.1 to 4.7)	.88
Change in pain VAS score	−11.7 ± 23.1	−11.6 ± 21.4	−0.1 (−7.1 to 6.8)	.97

a “Adhered” indicates patients who completed exercises at least 3 times per week in at least 9 of the first 12 weeks. HOS-ADL, Hip Outcome Score–Activities of Daily Living; iHOT-33, International Hip Outcome Tool–33; MD, mean difference; VAS, visual analog scale; PROM, patient-reported outcome measure.

b Data are presented as mean ± SD.

Post hoc subgroup analyses of outcomes demonstrated no significant heterogeneity of treatment effect between impingement types (Supplementary Tables 1 and 2).

There were no adverse events reported over the duration of the study.

## Discussion

In this study, we demonstrated that the HipFit10 program, a pelvic tilt–focused exercise regimen, successfully improves patient symptoms and helps patients avoid surgery at the 6-month follow-up. This provides further evidence to support consensus statements that all patients with FAIS should undergo a minimum period of 12 weeks of nonoperative management before considering hip arthroscopy.^23^ Engaging in an exercise-based pelvic tilt–focused protocol may allow patients to avoid being exposed to the risks and onerous recovery associated with surgery. With rates of hip arthroscopy increasing worldwide,^7,8,10,25,26^ this nonoperative intervention and approach have the ability to curb some of the increasing demand, personal health and system costs, and potentially morbidity associated with surgical care in this young active population.

Several previous studies have assessed the symptomatic improvement of patients with FAIS utilizing nonoperative treatment protocols, including 1 prospective cohort study by Pennock et al^39^ and 5 randomized trials comparing physical therapy–based programs to arthroscopic surgery.^17,20,28,29,35^ Similar to our findings, all studies demonstrated significant improvements in patient-reported outcomes from baseline to follow-up within the nonoperatively managed patients. However, direct comparison between our findings and these previously published studies does present some challenges due to differences in target populations, heterogeneity of interventions, and different outcome measures. The 5 randomized trials reported iHOT-33 change scores from baseline to 6 months^17,20,28,29^ or 8 months^35^ for patients treated nonoperatively, with group mean improvements of between 7.6 and 13.3 points across the studies. This range is comparable to our 6-month iHOT-33 change score of 11.7 points. In the Pennock et al^39^ cohort study of adolescent patients with FAIS, improvement was demonstrated using the modified Harris Hip Score from baseline to most recent scores (either at 12 or 24 months), with a mean difference of 20.1 points.

In terms of crossover to surgery, Pennock et al^39^ observed that 18% of their patients went on to surgery at the conclusion of the study. Two of the randomized studies^28,29^ also permitted crossover to surgery. Mansell et al^28^ randomized military patients with FAIS and observed a 70% crossover rate in those randomized to physical therapy. Similarly, Martin et al^29^ randomized patients over the age of 40 years with FAIS who had already engaged in a minimum of 8 weeks of physical therapy and observed a crossover rate of 68% among nonoperatively assigned patients. Each of these studies focused on a population that was quite different from ours, which may explain the discrepant crossover rates. In our study, we observed that 22% of patients opted to pursue surgery by 6 months, which is lower than the rates in the 2 randomized studies focused on adult patients, albeit at a shorter follow-up interval. Our findings strengthen what is shown in the existing literature that a nonoperative program for patients with FAIS significantly improves patient symptoms and can help a large proportion of individuals avoid surgery, at least in the short term.

To this point, the prescribed nonoperative regimens that have been published in these high-quality studies have varied widely between and within studies. A hallmark of the Pennock et al^39^ adolescent cohort study was rest and activity modification, with 59% of patients still avoiding return to sport at the conclusion of the study. In our protocol, patients were not restricted from sporting or exercise activity, and all patients were encouraged to return to their target sports within the study period. This is in accordance with research into patient goals of nonoperative FAIS treatment, where the most commonly expressed patient goals were activity based.^15^ All randomized trials used primarily in-person supervised and personalized physical therapy with limited direction as to the specific regimens delivered to the patients, resulting in significant heterogeneity. In the current study, we demonstrate similar magnitude of improvement among enrolled patients with a pelvic tilt–focused time-efficient exercise regimen that is feasible for patients to perform primarily on their own at home. The HipFit10 program may provide an avenue for patients with FAIS without supplemental health insurance benefits, or those who struggle to attend regular physical therapy due to access or time constraints, to significantly improve their symptoms and quality of life without incurring significant financial costs or personal time constraints.

In addition to improved patient function and quality of life, as measured by the iHOT-33, we also demonstrated a substantial improvement in pain VAS scores (11.7 points by 6 months), a secondary outcome. A reduction in pain has been shown to be a significant priority for patients with FAIS engaging in nonoperative care,^15^ and persistence of pain even in the setting of improved function may lead to pursuit of operative management. Of the aforementioned studies examining nonoperative treatment of FAIS, only that of Martin et al^29^ assessed pre- and posttreatment pain VAS scores. In their nonoperatively treated group, they found an improvement of 5.9 points by 6 months. While they did not present a comparison of the nonoperatively treated patients who crossed over to surgery and those who did not, a lack of improvement in pain could certainly be a potential explanation for the high crossover rate seen in their nonoperative arm. Future studies examining the role of pain and reduction in pain in success of nonoperative treatment in FAIS and helping patients avoid surgery could provide meaningful treatment insights that align with patient priorities.

Current consensus guidelines state that patients with FAIS should engage in 12 weeks of physical therapy–based nonoperative care before consideration for surgical management.^23^ However, in our study, we found that 18% of all enrolled patients experienced their significant improvement in a delayed fashion. Of the 105 patients with completed questionnaires who had not achieved the iHOT-33 MCID by 3 months, 34 (32%) of them went on to achieve the MCID by 6 months. This also represents one-third of the total patients who achieved the MCID threshold by 6 months. This finding calls into question whether 12 weeks is the most appropriate time threshold for a trial of nonoperative intervention before considering surgery, given that an additional one-third of patients who do not perceive benefit at 12 weeks may feel substantially better by 6 months. If we had stopped our treatment protocol at 12 weeks, it is likely that a meaningful proportion of patients would have pursued surgery who may have been able to avoid it with 6 months of exercise intervention. On the basis of these findings, surgeons and patients should evaluate whether an extended timeline of 6 months of nonoperative intervention ought to be attempted before considering surgical intervention in those hoping to avoid surgery.

Our findings were based on a pragmatic, intention-to-treat design with variable amounts of self-reported adherence between participants. Self-report measures may overestimate home exercise participation,^41^ yet alternative adherence measures often lack psychometric validity,^6^ and wearable tracking increases cost while reducing pragmatism and external validity.^9,12^ Thus, many studies, including ours, rely on self-reported adherence. In our cohort, 65% of patients reported adherence to the HipFit10 program, which is higher than typical musculoskeletal exercise adherence rates of 30% to 50%.^1,4,13,30,47^ This may reflect response or performance bias, although similar biases likely affected previous studies. Alternatively, higher adherence could relate to program features such as time efficiency, hybrid physical therapy sessions, or the relatively young athletic population. Despite this, nonadherence remained common, likely biasing results toward the null. Nevertheless, significant improvements in symptoms were observed at 3 and 6 months, with no significant difference between adherent and nonadherent groups. Future work examining adherence patterns may clarify dose-response relationships between utilization and outcomes.

What has not yet been established in the current literature is that there may be an archetype of a patient with FAIS who is more likely to benefit from a nonoperative FAIS program. Murphy et al^33^ attempted to identify factors baseline associated with success of nonooperative treatment success of FAIS based on the Australian FASHIoN Trial results; however, an inadequate sample size left the study underpowered to establish which baseline patient traits were associated with successful outcomes of nonoperative intervention.^33^ Future work to establish which baseline patient, clinical, and radiological factors are associated with improved outcomes with a modern nonoperative exercise regimen would be of significant value in determining who best to enroll in a similar program.

### Limitations

Despite its strengths, this study has several limitations. First, this study focused on a relatively short-term outcome of 6 months to assess the direct influence of the exercise program on symptoms and patient treatment decisions. Accordingly, we are unable to determine the long-term effect of the exercise program on the avoidance of surgery and how improvements may or may not be sustained after participants stop performing the exercises. Future studies examining the durability of similar initial treatment protocols are needed. Second, this is a prospective cohort focusing exclusively on the outcomes of those treated with the exercise program, and there is no control group. As with all home-based exercise interventions, adherence likely played a significant role in the likelihood of achieving outcomes. While self-reported adherence was tracked throughout the program, this may be subject to reporting bias. Taking a pragmatic approach, we did not exclude from analysis patients who did not adhere to the program or conduct any objective measures of tracking adherence beyond self-reporting. Third, the population included in this study is a relatively active young population who were seen at an academic center, and thus the findings may not be generalizable to populations who do not fit these descriptions. Additionally, participants in this study were willing to enroll in a nonoperative treatment program, which introduced the potential for selection bias in favor of a successful outcome. Therefore, these results may not apply to patients strongly favoring operative treatment at the time of initial consultation. Fourth, we applied a relatively minimalist and pragmatic intervention of a home-based exercise program focusing on pelvic tilt. Accordingly, the findings of this study may be less applicable to clinicians aiming to prescribe a more comprehensive and controlled nonoperative protocol that includes medications, prescribed activity modification, injections, and other interventions.

## Conclusion

In this study of 214 patients with FAIS, pelvic tilt–focused physical therapy was utilized to help the majority of participants avoid surgery. Patients improved significantly over the duration of the program in terms of their functional scores. On the basis of the results of this study, the HipFit10 program can be recommended as a safe and effective nonoperative treatment option for patients with FAIS.

## Supplemental Material

sj-docx-1-ajs-10.1177_03635465261454648 – Supplemental material for Treatment of Patients With Femoroacetabular Impingement Syndrome Using a Pelvic Tilt–Focused Exercise Program: A Prospective Cohort Supplemental material, sj-docx-1-ajs-10.1177_03635465261454648 for Treatment of Patients With Femoroacetabular Impingement Syndrome Using a Pelvic Tilt–Focused Exercise Program: A Prospective Cohort by Graeme Hoit, Daniel B. Whelan, Tim Dwyer, Sheilah Hogg-Johnson, Jaskarndip Chahal and Aileen M. Davis in The American Journal of Sports Medicine

sj-docx-2-ajs-10.1177_03635465261454648 – Supplemental material for Treatment of Patients With Femoroacetabular Impingement Syndrome Using a Pelvic Tilt–Focused Exercise Program: A Prospective Cohort Supplemental material, sj-docx-2-ajs-10.1177_03635465261454648 for Treatment of Patients With Femoroacetabular Impingement Syndrome Using a Pelvic Tilt–Focused Exercise Program: A Prospective Cohort by Graeme Hoit, Daniel B. Whelan, Tim Dwyer, Sheilah Hogg-Johnson, Jaskarndip Chahal and Aileen M. Davis in The American Journal of Sports Medicine
