# Supplementary material for: Treatment of Patients With Femoroacetabular Impingement Syndrome Using a Pelvic Tilt–Focused Exercise Program: A Prospective Cohort
Source: Am J Sports Med. 2026 Jun 17;54(9):2166–77. doi: 10.1177/03635465261454648 (PMC13354838; doi:10.1177/03635465261454648)
Supplement: sj-docx-1-ajs-10.1177_03635465261454648 – Supplemental material for Treatment of Patients With Femoroacetabular Impingement Syndrome Using a Pelvic Tilt–Focused Exercise Program: A Prospective Cohort [file sj-docx-1-ajs-10.1177_03635465261454648.docx]

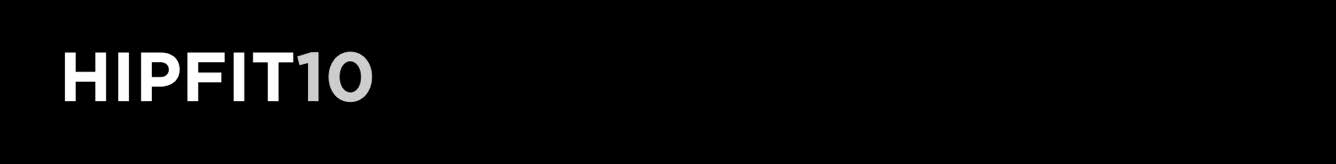


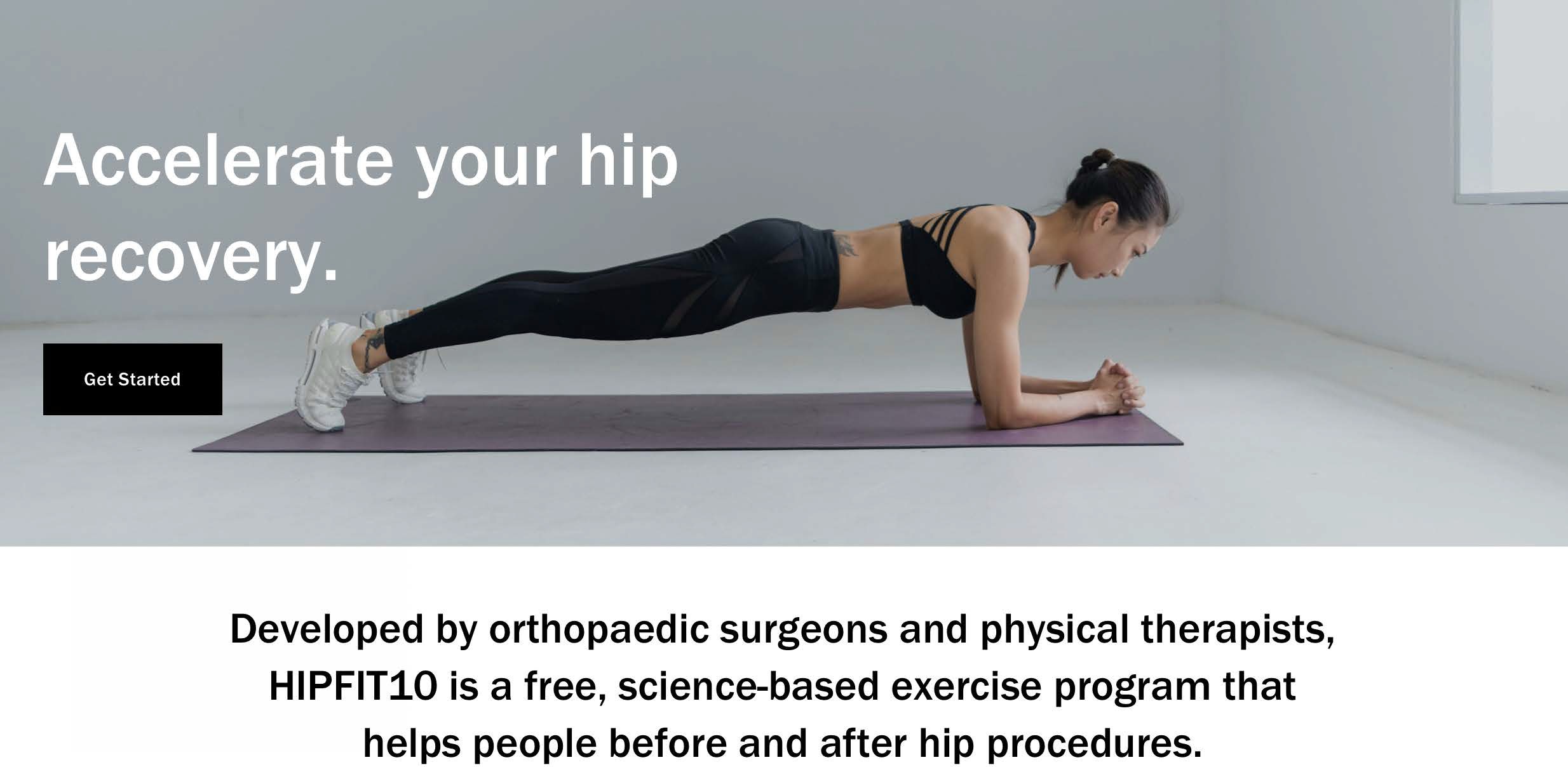


Visit [www.hipfit10.com](http://www.hipfit10.com/) for the complete program and more information


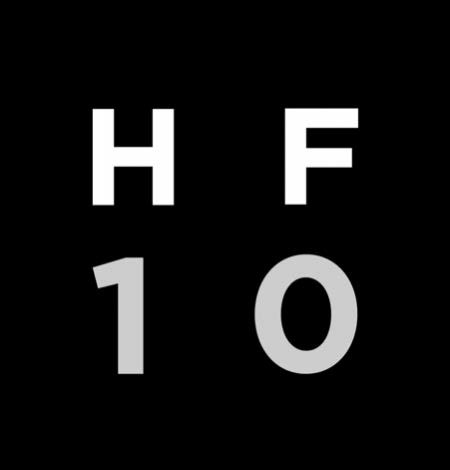


HIPFIT10 Exercise Program Instructions

- Complete exercises 4 to 5 times per week at your availability.
- Starting with exercise 1, do as many repetitions of exercise in 45 seconds as you are able. Take as many rests as you need.
- Some of the exercises will be “easy”, like exercise 1. These work on postural control and small movements.
- Some of the exercises will be “hard”, like exercise 4. These work on strengthening and power.
- If you are unable to do two consecutive repetitions without rest, apply the “Beginner ModiGcation” if there is one, or discuss with your physiotherapist on ways you can modify the exercise.
- If you are able to do an exercise without rest for the full 45 seconds three days in a row, apply the “Extra Challenge” if you are able.
- On off days, try a stretching protocol that focuses on your hip Jexors, quads and hamstrings and that keeps you out of positions that make your hip hurt. DO NOT do any stretches that make your hip hurt more. Try what works for you. If you Gnd that stretching does not help you, use your off days for rest and recovery.


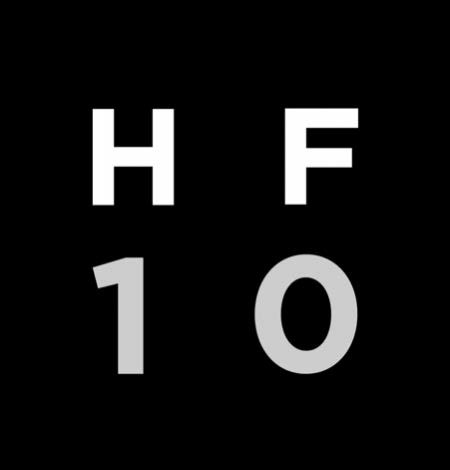


#
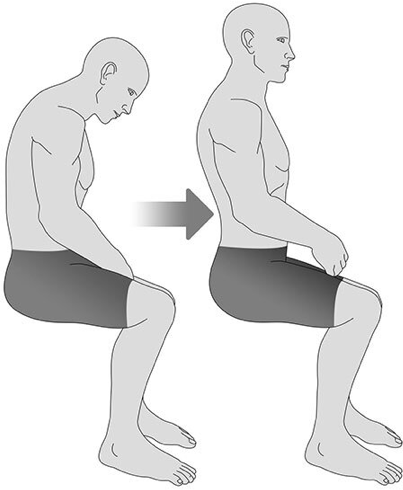
Exercise 1: Seated Pelvic Tilts

- - Sit at edge of chair
  - Relax abdominal and pelvic muscles
  - Tighten lower back and pelvic muscles to exaggerate the lumbar curvature of the lower back into an upright posture

***Extra Challenge:*** try it while seated on an exercise ball

# Exercise 2: Straight Leg Raises


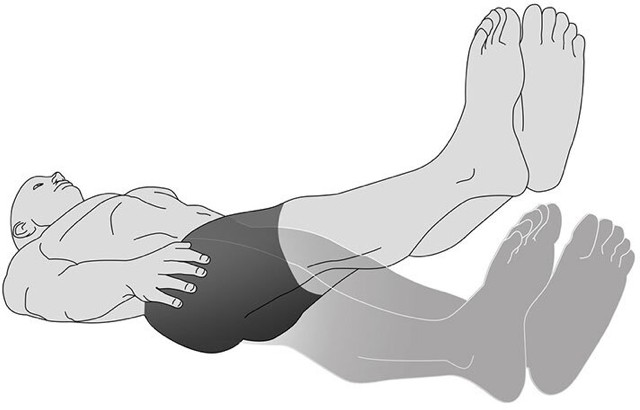
Lying on your back, with your hands at your sides, raise both feet 2-6 inches off the ground. Lower them back down without touching the ground and repeat as many times as you can in 45 seconds.

Rest as necessary.


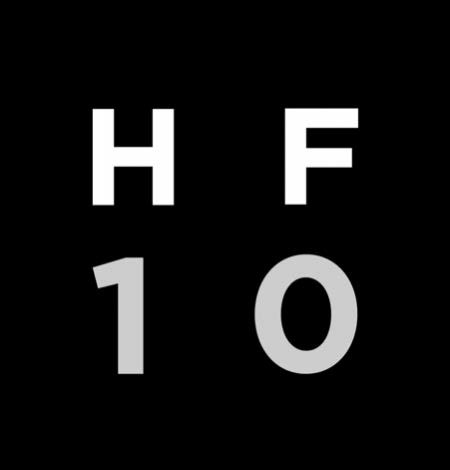
***Beginner modi3cation:*** Have a friend hold the bottom of your heels and take off enough weight so that you are able to do three repetitions without rest.

#
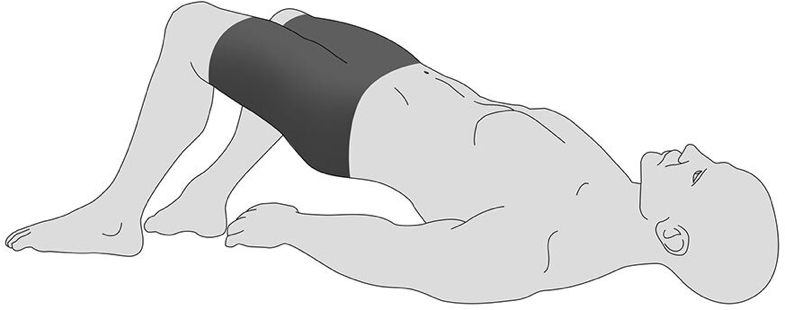
Exercise 3: Bridges

Lie on your back with your hip and knees bent. Lift your hips but keep your shoulders on the ground. Lower yourself to the ground, then repeat for 45 seconds.

# Exercise 4: Flutter Kicks


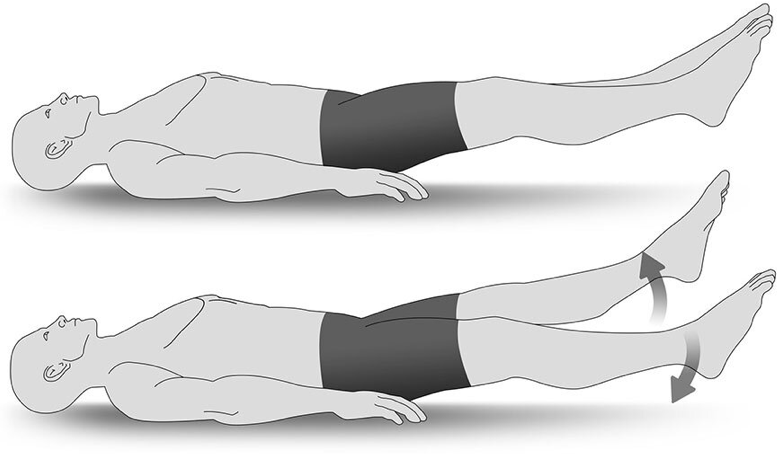
Lying on your back with your hands at your sides, lift your feet off the ground.

Alternate lifting your feet 2-6 inches in the air without letting either foot touch the ground. Complete for 45 seconds, rest as necessary.

***Extra Challenge:*** put on ankle weights

***Beginner modi3cation:*** Have a friend hold the bottom of your heels and take off enough weight so that you are able to do three repetitions without rest.

#
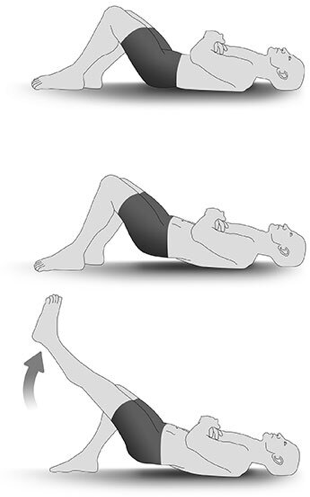
Exercise 5: Supine Leg Lifts

Lying on your back with your knees bent and feet slightly apart. Tighten your lower stomach and lift your pelvis off the ground slightly. Straighten one of your legs and hold this position for 5 seconds, then alternate as many times as possible in 45 seconds.

#
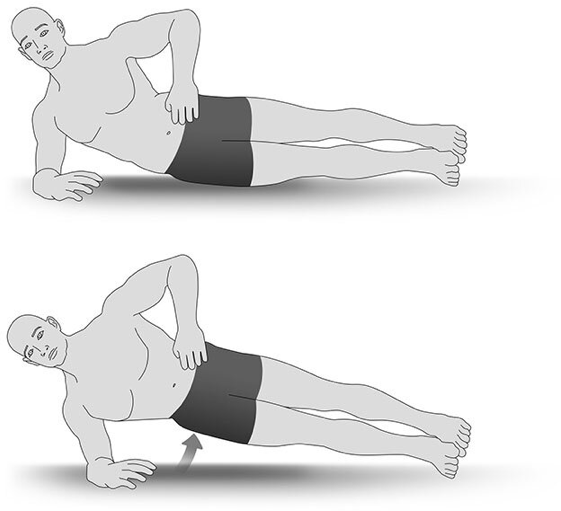
Exercise 6: Side Plank Hip Lifts

Lie on your side with your bottom elbow on the ground, and your top hand on your top hip. Lift your bottom hip off the ground as many times as possible in 20 seconds, then switch sides and repeat for 20 more seconds.

***Extra challenge:*** hold a dumbbell weight in your top hand

#
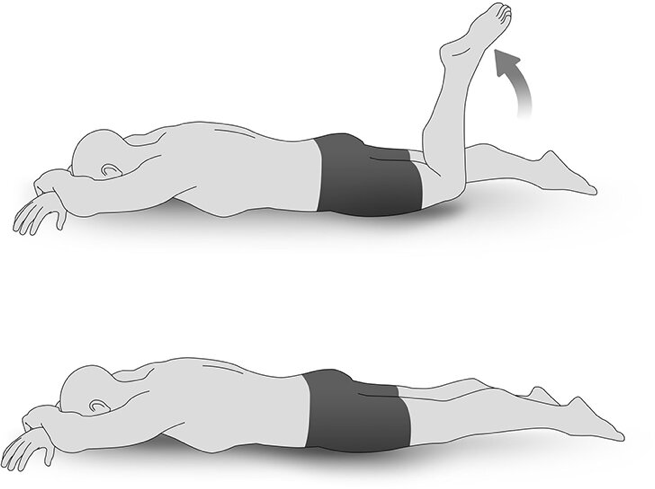
Exercise 7: Prone Single Leg Lifts

Lie face down with your hips and back relaxed and one knee bent. Tighten the lower stomach, squeeze your buttock together and lift the bent leg 3 inches off the ground. Hold this for 5 seconds.

Alternate sides as many times as possible in 45 seconds.

# Exercise 8: Bird-Dog


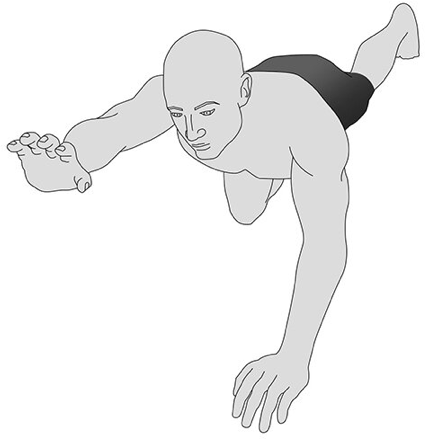
Position yourself on your hands and knees with your head looking forward. Lift and extend your opposite arm and leg, keeping your back and core tight. Hold for 5 seconds and alternate sides as many times as possible in 45 seconds.

***Extra Challenge:*** Touch your opposite elbow and knee together when switching from side to side.

#
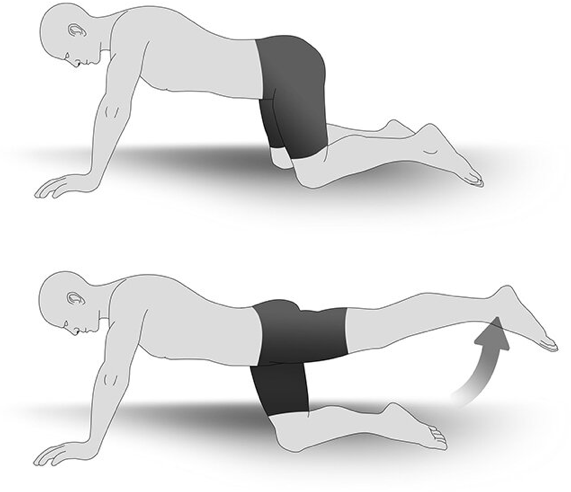
Exercise 9: Leg Extensions


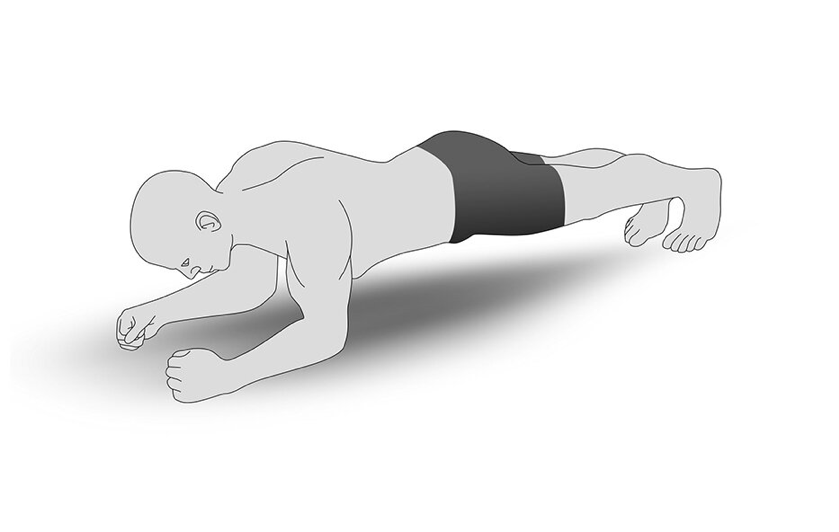


**Exercise 10:**

**Forearm Plank**

Position yourself on your forearms and toes, with your body straight. Hold this pose for as long as possible during the 45

second timer. Rest in necessary.

***Extra challenge:*** crunch your abs for 5

Position yourself on your hands and knees. With the core tight, slowly straighten one leg behind you, contracting your buttocks. Hold for 5 seconds then slowly lower.

Alternate sides as many times as possible

in 45 seconds.

seconds on, 5 seconds off during exercise

# Off-Day Stretches

To keep from tightening up, perform each of these stretches for 30 seconds. Repeat for three rounds.

**Hip Flexors Hamstrings Quads**

**
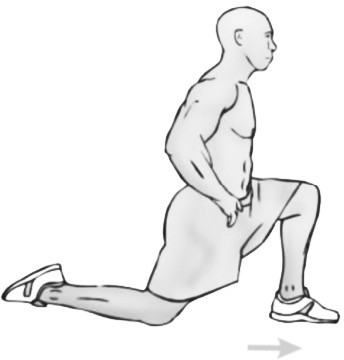

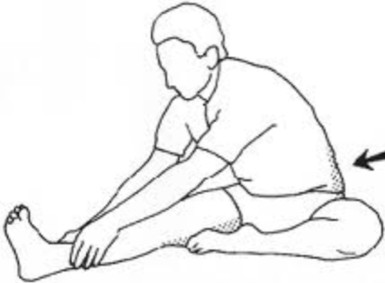

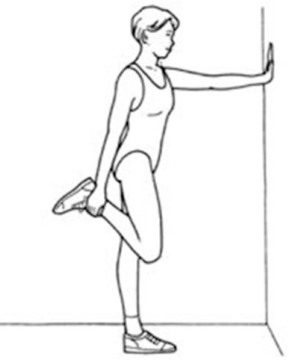
**

Visit [www.hipfit10.com](http://www.hipfit10.com/) for the complete program and more information
