# Supplementary material for: Treatment of Patients With Femoroacetabular Impingement Syndrome Using a Pelvic Tilt–Focused Exercise Program: A Prospective Cohort
Source: Am J Sports Med. 2026 Jun 17;54(9):2166–77. doi: 10.1177/03635465261454648 (PMC13354838; doi:10.1177/03635465261454648)
Supplement: sj-docx-2-ajs-10.1177_03635465261454648 – Supplemental material for Treatment of Patients With Femoroacetabular Impingement Syndrome Using a Pelvic Tilt–Focused Exercise Program: A Prospective Cohort [file sj-docx-2-ajs-10.1177_03635465261454648.docx]

**Supplementary Table 1**: Post-hoc subgroup analysis of improvements in patient reported outcome measures by impingement type.

| **Time Period** | **Change Score** | **Cam** | **Pincer** | **Mixed** | **p-value*** |
| --- | --- | --- | --- | --- | --- |
| **0-3 months** | iHOT-33, mean difference (+SD) | 8.9 (12.4) | 8.3 (14.2) | 5.6 (14.4) | 0.32 |
|  | HOS-ADL, mean difference (+SD) | 4.4 (13.1) | 2.4 (11.8) | 0.8 (13.2) | 0.24 |
|  | Pain VAS, mean difference (+SD) | -7.6 (18.5) | -11.1 (21.0) | -5.0 (21.0) | 0.27 |
| **0-6 months** | iHOT-33, mean difference (+SD) | 12.1 (16.4) | 11.7 (16.4) | 11.1 (18.5) | 0.94 |
|  | HOS-ADL, mean difference (+SD) | 5.0 (16.0) | 3.9 (12.1) | 3.4 (13.3) | 0.80 |
|  | Pain VAS, mean difference (+SD) | -12.9 (23.2) | -11.9 (20.0) | -10.2 (23.6) | 0.78 |

*p-values generated using one-way analysis of variance.

Abbreviations: iHOT-33: International Hip Outcome Tool-33, SD: standard deviation, HOS-ADL: Hip Outcome Score – Activities of Daily Living, VAS: visual analogue scale.

**Supplementary Table 2**: Post-hoc subgroup analysis of outcomes by impingement type.

| **Outcome** | **Cam**  **n=77** | **Pincer**  **N=49** | **Mixed**  **N=66** | **p-value*** |
| --- | --- | --- | --- | --- |
| Achieved 6-month iHOT-33 MCID, N (%) | 44 (57%) | 23 (47%) | 36 (54%) | 0.30 |
| Avoided Surgery, N (%) | 59 (77%) | 39 (79%) | 55 (83%) | 0.63 |

*p-values generated using chi-square.

Abbreviations: N: number of patients, iHOT-33: International Hip Outcome Tool-33.
